# Portrayal of risk information and its impact on audiences’ risk perception during the Covid‐19 pandemic: A multi‐method approach

**DOI:** 10.1111/risa.17681

**Published:** 2024-11-20

**Authors:** Annemarie Wiedicke, Paula Stehr, Constanze Rossmann

**Affiliations:** ^1^ Institute for Media Research Chemnitz University of Technology Chemnitz Germany; ^2^ Depatment of Media and Communication LMU Munich Munich Germany

**Keywords:** Covid‐19 pandemic, framing, risk information, risk perception, SARF

## Abstract

Over the last years, infectious diseases have been traveling across international borders faster than ever before, resulting in major public health crises such as the Covid‐19 pandemic. Given the rapid changes and unknown risks that mark such events, risk communication faces the challenge to raise awareness and concern among the public without creating panic. Drawing on the social amplification of risk framework—a concept that theorizes how and why risks are amplified or attenuated during the (1) transfer of risk information (by, for instance, news media) and (2) audiences’ interpretation and perception of these information—we were interested in the portrayal of risk information and its impact on audiences’ risk perception over the first wave of the Covid‐19 pandemic in Germany. We therefore conducted a quantitative content analysis of a major public and private television (TV) newscast (*N* = 321) and combined it with survey data (two‐wave panel survey, t1: *N* = 1378 and t2: *N* = 1061). Our results indicate that TV news (as a major information source at that time) were characterized by both risk‐attenuating and risk‐amplifying characteristics, although risk‐amplifying attributes were particularly pronounced by the private TV newscast. Notably, those who only used private TV news between both waves showed the highest perceived severity at time 2. However, the interaction effect of time and use of public and/or private TV news was only significant for perceived susceptibility. Overall, more research is needed to examine the effects of different types of media and changes in risk perceptions over time.

## 1 INTRODUCTION

Over the last years, infectious diseases have been traveling across international borders faster than ever before (Chon & Park, 2019). This has not only led to major public health crises, such as the recent Covid‐19 pandemic (Tandoc & Lee, 2022), but has also drawn increasing attention to crisis communication and its audiences. Given the rapid changes, high uncertainties, and major damages potentially caused in public health crises and pandemics (Link, 2021; Tang & Zou, 2021), communication plays a major role during such events: Accurate and timely information help the public understand the situation and cope with its uncertainties. Moreover, it empowers individuals to make informed decisions about preventive behaviors (Link, 2021; Tandoc & Lee, 2022). However, at the same time, crisis communication faces the challenge to raise awareness and concern among the public without creating panic or inducing irrational behavior (Rossmann et al., 2018; Seeger et al., 2010). In consequence, enabling the public to develop adequate (or accurate) risk perceptions is a major function of crisis communication (Rossmann et al., 2018): People with adequate risk perceptions neither underestimate nor overestimate risks arising during public health crises and pandemics. Importantly, both underestimation (which might lead to risky behaviors potentially harming individuals and society at large) and overestimation (which might increase distress and anxiety) have been shown to present additional challenges to managing public health crises (Sinclair et al., 2021). Here, the mass media, particularly news media, play a significant role, as they have been frequently shown to affect audiences’ risk perception (e.g., Chung & Jones‐Jang, 2022; Vanherle et al., 2023; Zhao & Wu, 2021).

Previous research in this context often draws on the *social amplification of risk framework* (SARF) (Kasperson et al., 1988), which theorizes how and why risks or risk events are amplified or attenuated (Rossmann et al., 2018). Specifically, the framework assumes amplification (or attenuation) processes at two stages (Kasperson et al., 1988)—first, the transfer of information about the risk (such as the communication of risks by news coverage) and, second, the response mechanisms of society (in other words, audiences’ interpretation and perception). Due to their important role in transferring information during health crises, the mass media play a crucial role as stations of information transfer (Rossmann et al., 2018). However, current evidence on the way in which risk information have been portrayed in the news coverage of the Covid‐19 pandemic is inconclusive. In particular, research systematically analyzing characteristics of media content that—drawing on the SARF—may attenuate or amplify risks is still missing. As for the (news) media's impact on audiences’ risk perception during the crisis, current research also provides conflicting results (Cipolletta et al., 2022), as some studies indicate an amplification of risks, whereas others suggest attenuation processes. Finally, prior studies in the SARF context analyze *either* the communication about risks *or* audiences’ responses to risk communication. Therefore, they lack an integrative perspective.

Thus, we chose to address both aspects in our study: On the one hand, we asked to what extent German television (TV) news—as one of the most important Covid‐19‐related information sources in Germany (Bendau et al., 2021; Dreisiebner et al., 2022), the use of which particularly increased within the first weeks of the pandemic (Dan & Brosius, 2021)—exhibited characteristics that are considered to enhance risk perceptions (such as the volume of information or its dramatization, Rossmann et al., 2018). On the other hand, we were interested in the association between TV news use and changes in risk perception in Germany during the Covid‐19 pandemic. Following a multi‐method approach, we provide first insights into the complex relations between the characteristics of TV news in times of crisis and their impact on audiences’ risk perceptions.

## 2 THE MEDIA'S ROLE DURING PUBLIC HEALTH CRISES

### 2.1 Crisis communication and risk perception

Correct and up‐to‐date information is of major significance during a public health crisis such as the Covid‐19 pandemic, which—due to more than 774 million confirmed cases and more than 7 million deaths worldwide (February 2024)—can be characterized as the most significant public health event of the past century (WHO, 2024). In these situations, public communication mainly serves three functions. These include (1) providing information about risks, symptoms, or treatment of a disease; (2) encouraging the public to take preventive action and adhere to healthcare and political and social interventions; and, finally, (3) allowing the public to evaluate risks and deal with the threats appropriately (Rossmann et al., 2018, p. 358). Notably, there is an increasing body of research pointing out shortcomings of the communicative measures of public health authorities and governments during the Covid‐19 pandemic, such as a lack of coherency and clarity (e.g., Alami et al., 2021; Cernicova‐Buca & Palea, 2021; Sauer et al., 2021; Wild et al., 2023).

Importantly, good crisis communication is also relevant in situations where crisis management is largely based on legal obligations or restrictions—which was the case in many countries during the Covid‐19 pandemic, including Germany (e.g., the duty to wear masks, lockdown). Drawing on the self‐determination theory (Deci & Ryan, 2000), previous studies have shown that autonomy and intrinsic motivation increased the readiness to engage in Covid‐19 protection measures, whereas a perceived lack of autonomy—caused, for instance, by legal obligations—led to frustration and a lower protection motivation (Morbée et al., 2021; Porat et al., 2021). However, the perception of autonomy is not only a consequence of the legal restrictions themselves but also of the way in which these restrictions are communicated. Accordingly, Legate and Weinstein (2022) have found that people were more motivated to engage in protective behaviors during the Covid‐19 pandemic if they perceived the communication style as autonomy‐supportive (e.g., clear reasoning, emphasis on several options, enabling informed decisions; also see Martela et al., 2021; Spisak & McNulty, 2023).

So, overall, good crisis communication is of great significance during a global public health event such as the Covid‐19 pandemic for a variety of reasons. In our study, we chose to focus on the way in which crisis communication has affected people's risk perceptions (Rossmann et al., 2018; Seeger et al., 2010). *Risk perception* is a multidimensional concept, and it entails referential, cognitive, affective, and behavioral components (Coleman, 1993). In the context of public health crises and pandemics, risk perception has been referred to as a person's subjective assessment of the actual or potential threat to their life or, more broadly, their well‐being (Lohiniva et al., 2022). More specifically, it comprises two dimensions, namely, the *perceived susceptibility* (or, in other words, the probability of being infected with SARS‐CoV‐2) and the *severity of potential consequences* (Hubner & Hovick, 2020). Importantly, past crises have shown that the success of policies to slow down the spread of infectious diseases depends, at least partly, on the public's recognition of the according risks (Slovic, 2000; Zhou, 2022). People who perceive greater risks are more likely to adopt preventive behaviors (Bruine de Bruin & Bennett, 2020; Dryhurst et al., 2020) and accept measures implemented by the government (Siegrist et al., 2021). At the same time, higher risk perception has also been shown to negatively affect people's psychological states (Cipolletta et al., 2022; Lam et al., 2020; Zhong et al., 2021). Lower risk perception, however, may prevent people from adopting preventive behaviors (Cipolletta et al., 2022; Wright, 2021). In consequence, establishing adequate risk perceptions among audiences is of great significance for the success of communication efforts in a public health crisis (Dryhurst et al., 2020; Zhou, 2022).

Notably, audiences’ perception of risks during public health crises and pandemics depends on various factors (Zhou, 2022). According to findings from the Covid‐19 pandemic, these include, for instance, personal experience with the virus; hearing about the virus from friends and family; trust in government, science, and medical professionals; values and worldviews; as well as knowledge of the government's strategy (Botzen et al., 2022; Dryhurst et al., 2020; Ju & You, 2022; Schneider et al., 2021). Moreover, mass media are considered to be vital shapers of the public's risk perceptions (Oh et al., 2021; Snyder & Rouse, 1995). This applies especially to situations in which individuals lack first‐hand experience of a particular health threat (Oh et al., 2015)—such as knowledge about Covid‐19 during the early stages of the pandemic. Therefore, we consider mass media to be an important source of risk information in our context.

### 2.2 (News) media and the social amplification of risk

By drawing on psychological, sociological, and cultural perspectives of risk perception as well as risk‐related behavior, the *social amplification of risk framework*—short SARF (Kasperson et al., 1988)—theorizes how and why risks or risk events are amplified, or, on the contrary, attenuated. In particular, SARF assumes amplification or attenuation processes at two stages of the information flow: first, the transfer of information about the risk, and second, the response mechanisms of audiences and society (Kasperson et al., 1988; Rossmann et al., 2018). As amplification, respectively, attenuation stations, Kasperson et al. (1988) name both individuals and societal actors or institutions, including, for example, laymen, scientists who communicate risk assessments, social groups and opinion leaders within these groups, as well as (news) media.

Especially when direct experience with a health threat is (still) lacking (Oh et al., 2015), information shared via (news) media is considered to become “the central agent of public risk perception” (Rossmann et al., 2018, p. 359): Individuals learn about a particular risk through media that—although providing the risk messages—also interpret the risk issues (Oh et al., 2021). Consequently, the media process and depict information in a way that shapes the salience of the communicated risk that in turn may affect people's risk perception (Chong & Choy, 2018).

According to the SARF, there are several attributes of information that may affect the amplification process (Kasperson et al., 1988; Rossmann et al., 2018). First, early studies in this context have shown that the (1) *volume of information* (the quantity of news coverage or, in more general terms, the media attention) alone can affect risk perceptions regardless of the content presented (Mazur, 1984, 1990). In consequence, it can be assumed the quantity of news coverage (or other media contents) may be at least one factor contributing to societal amplification or attenuation processes during a public health crisis (Kasperson et al., 1988; Rossmann et al., 2018). That means a higher volume of coverage may lead to higher risk perceptions, whereas a low volume of information may attenuate risk perceptions. In addition, the (2) *degree of debates or controversy*, such as disputes among experts and authorities regarding the best way to slow down the spread of an infectious disease, may also increase public risk perception (Liu et al., 2008; Vries et al., 2021). This holds true for (3) *dramatization* as well. There are several ways in which news coverage or other media may amplify risks during a public health crisis, including the emphasis of negative consequences, such as illness and death, as well as the use of emotional and alarming language (Rossmann et al., 2018).

Finally, the media's use of certain (4) *symbolic connotations* is also assumed to have an impact on their recipients’ risk perceptions. Originally, symbols have been described as “specific terms or concepts used in risk information” (Kasperson et al., 1988, p. 185), which may have different meanings among different social and cultural groups, and potentially trigger associations that differ from those intended. Notably, more recent publications also refer to the media's *framing of risks* as significant symbols in this context (e.g., Kasperson et al., 2003; Rossmann et al., 2018).

In particular, the news coverage of epidemics and pandemics such as the Covid‐19 pandemic is mainly characterized by five frames (Rossmann et al., 2018; Shih et al., 2008)—*action*, *reassurance*, *new evidence*, *uncertainty*, and *consequences* (including the specific consequences discussed). Although the first three frames are considered *risk‐attenuating*, the emphasis of uncertainty and negative consequences of a pandemic is assumed to be *risk‐amplifying* (ibid.). Table 1 offers an overview of the different frames and their meaning.^1^

**TABLE 1:** Potentially risk‐attenuating and risk‐amplifying frames in news coverage on epidemics and pandemics.

Risk‐attenuating	**Action** … describes all measures taken against the dissemination of a virus such as SARS‐CoV‐2, including, for instance, medical measures or political strategies
	**Reassurance** … emphasizes the idea that the public should not worry about the pandemic or its impact
	**New evidence** … refers to new evidence helping to understand SARS‐CoV‐2 (or similar), reduce its spread, or quell the infection
Risk‐amplifying	**Uncertainty** … describes the portrayal of uncertainties in any aspect(s) of the pandemic, including the cause, the cure, the possible spread, or similar
	**Consequences** … addresses the dissemination of a virus such as SARS‐CoV‐2 and/or its individual, social, political, or economic consequences **Consequences in detail** … refers to the specification of consequences addressed in the news item

Notably, a considerable number of studies have addressed the way in which news coverage has portrayed risk information throughout the Covid‐19 pandemic—and whether these portrayals did contain information that could be, according to the criteria described above, considered risk‐amplifying. First, there has been continuous and intense news coverage on the Covid‐19 pandemic (Dreisiebner et al., 2022) and thus a potentially risk‐amplifying high (1) *volume of information*. For instance, an automatic analysis of more than 26 million news articles from 172 news sources in eleven countries indicates that, especially in the first months of the pandemic in 2020, news coverage worldwide has been greatly dominated by this issue (Krawczyk et al., 2021). As for the (2) *degree of debates or controversy*, the evidence is less clear: Hart et al. (2020) found the US news coverage on Covid‐19 to be highly polarizing, whereas print media in China rarely depicted conflicts among experts or similar (Owusu Ansah et al., 2021). In contrast, results from Germany indicate that—although low at first—the degree of debates depicted in German news coverage increased over the course of the pandemic (Maurer et al., 2021). With regards to (3) *dramatization* through emotional or alarming language, there is only limited evidence. A comparative content analysis of online news headlines in March 2020 in Italy, the United States, and South Africa (as countries with particularly high infection rates) shows that headlines often contained numbers of deaths and that the Covid‐19 pandemic has been portrayed as “a lethal pandemic that destroys and disrupts human life” (Ebrahim, 2022, p. 1), or, in other words, highly emotional. Similarly, a content analysis of Covid‐19 coverage in Israeli ultra‐orthodox religious media between January and March 2020 indicates a high proportion of articles containing emotional and alarming language, with emotionally charged words often being positioned in the headline or the subtitle (Gering & Cohen, 2023).

Finally, with regards to the (4) *use of specific media frames*, a comparative content analysis of the Covid‐19 news coverage in Argentina, Germany, Pakistan, South Africa, South Korea, and the United States indicates a dominance of both the (potentially risk‐amplifying) *consequences* and the (potentially risk‐attenuating) *action* frame (Bhatti et al., 2022). In contrast, the *uncertainty* (risk‐amplifying), *reassurance*, and *new evidence* (both risk‐attenuating) frames have been used less frequently (Bhatti et al., 2022). Interestingly, and in line with evidence from prior epidemics and pandemics (e.g., Fung et al., 2011; Rossmann et al., 2018; Sandell et al., 2013), current content analyses point out not only cultural (Bhatti et al., 2022) but also media‐related differences when it comes to the portrayal of risk information in news coverage. For instance, findings from Italy indicate that public TV channels, compared to private TV channels, reported less on Covid‐19 and put more emphasis on measures taken against the further dissemination of the virus, that is, action (Miconi et al., 2023).

In conclusion, there is evidence that Covid‐19 news coverage has, overall, been characterized by both risk‐attenuating and risk‐amplifying attributes. However, current evidence is hardly systematic. Thus, to our knowledge, an analysis of news coverage about the Covid‐19 pandemic—despite it being the most significant public health event of the past century (WHO, 2023)—that explicitly draws on the SARF and examines all content characteristics that may shape risk perceptions is still missing.

As for audiences’ responses to media's risk communication, current research provides conflicting results (Cipolletta et al., 2022). On the one hand, *amplification* processes have been repeatedly identified. For instance, social media have been pointed out as amplification stations during various public health crises and pandemics (e.g., Ali et al., 2019; Oh et al., 2021), including the Covid‐19 pandemic in China (Zhao & Wu, 2021; Zhou, 2022). Moreover, a daily diary study from Belgium (March and April 2021) revealed that the reception of news media (but not social media) increased risk perceptions regarding Covid‐19 among participants (Vanherle et al., 2023). This is confirmed by yet another study from China in which news media were found to be significant for amplifying audiences’ risk perceptions (Zhao & Wu, 2021).

On the other hand, there is evidence of risk *attenuation* processes. For instance, a study by Zhang and Cozma (2022) points out the risk‐attenuating role of Twitter during the early stages of the Covid‐19 pandemic in the United States, whereas Chung and Jones‐Jang (2022) found similar effects as a result of obtaining Covid‐19‐related information from conservative media and Trump briefings. However, according to current research by Nazione et al. (2021), exposure to neither news media nor social media did significantly predict risk perceptions during the early stages of the Covid‐19 pandemic in the United States.

Consequently, more (longitudinal) research is needed to understand how the use of specific information channels and audiences’ risk perceptions are related. Besides, as laid out above, prior studies in the SARF context tend to analyze *either* the communication about risks *or* audiences’ responses to risk communication. Therefore, an integrative perspective linking results of media content analyses with public survey data is currently still missing.

## 3 RESEARCH QUESTIONS

Against this backdrop, our study addresses several gaps in literature. First, we aim to empirically explore the TV news coverage on Covid‐19 and the relation between people's TV news use and their risk perceptions. Because news media are assumed to shape the public's risk perceptions particularly in times in which people (still) lack first‐hand experience of a particular health hazard (Oh et al., 2015), we analyze the TV news coverage during the first wave of the Covid‐19 pandemic in Germany in March and April 2020. At that time, numbers of confirmed cases and especially Covid‐19‐related deaths were still comparatively low in Germany. For instance, as of April 30 in 2020, Germany reported 159,119 cases and 6288 deaths, whereas Italy and the United States reported 203,591 cases and 27,682 deaths, respectively, 1003,974 cases and 52,428 deaths (WHO, 2020). Still, both case numbers and the amount of Covid‐19‐related deaths had grown considerably since the first officially known infection on January 27 (BMG, 2023). Therefore, on March 22, Germany entered its first lockdown, which lasted until May 4 (Thurau & Bosen, 2021). In our analysis, we focus on TV news, given their role as major information source at the beginning of the pandemic in Germany (Bendau et al., 2021; Dan & Brosius, 2021; Dreisiebner et al., 2022). Thus, we ask:

RQ1: *To what extent does German TV news coverage exhibit characteristics that may have amplified or attenuated recipients’ risk perceptions?*

Previous studies have identified media‐specific differences in the portrayal of risk information (Bhatti et al., 2022; Rossmann et al., 2018; Sandell et al., 2013), including differences between public and private TV newscasts in Italy regarding their depiction of Covid‐19 (Miconi et al., 2023). In Germany, there is also a dual broadcasting system with private and public TV channels. Public TV channels are, to a large amount, financed without advertising and were institutionalized in Germany after the Second World War (based on the model of the BBC). To this day, they are legally responsible for providing information that enable the formation of a public opinion, to “fulfill the democratic, social and cultural needs of society.” Moreover, they are obligated to offer “education, information, advice, and entertainment” (Bundeszentrale für politische Bildung, 2024a). Private TV channels, in contrast, do not have such legal obligations and are mainly financed through advertising (Bundeszentrale für politische Bildung, 2024b). So, overall, public and private TVs in Germany differ regarding their organizational structure, legal responsibilities, and funding (Beck, 2018). Against this backdrop, we were interested in whether they showed dissimilarities in their depiction of risk information, therefore asking:

RQ2: *Do public and private TV news differ in their portrayal of risk information?*

As indicated above, current research provides conflicting results regarding the news media's impact on audiences’ risk perceptions (e.g., Chung & Jones‐Jang, 2022; Nazione et al., 2021; Vanherle et al., 2023), indicating both culture‐ and media‐specific differences. Moreover, evidence from Germany is lacking. Thus, our study addresses the following research question:

RQ3: *Do people (not) using public and/or private TV news differ in their risk perceptions?*

Previous studies only examine cross‐sectional relations between the use of different types of media and Covid‐19‐related risk perception (e.g., Karasneh et al., 2021; Shen & Yang, 2022; Zeballos Rivas et al., 2021). However, to draw causal conclusions regarding the role of media use in shaping risk perceptions, a longitudinal perspective is needed. Hence, we examine the following question:

RQ4: *Can changes in risk perception over time be explained by (not) using public and/or private TV news?*

To answer these questions, we followed a multi‐method approach. More specifically, we combined a quantitative content analysis of German TV news coverage addressing RQ1 and RQ2 with a panel survey among the German population addressing RQ3 and RQ4. Data collection took place during the first wave of the Covid‐19 pandemic in Germany. Both studies are laid out in detail below.

## 4 STUDY 1: QUANTITATIVE CONTENT ANALYSIS

### 4.1 Methods

First, we examined the Covid‐19‐related reporting of leading German TV news (NDR, 2021; RND, 2020)—*Tagesschau* (public TV newscast) and *RTL Aktuell* (private TV newscast)—between March 1 and April 22, 2020. We chose to focus on these newscasts specifically, as they have shown the highest viewing figures among German public and private newscasts for years, with market shares of 39.5% (*Tagesschau*) and 15.0% (*RTL Aktuell*) in 2020 (Haddad et al., 2023). To reduce the sampling material, every second newscast (*N *= 54) was selected for coding. For each newscast, we noted its overall number of news items but only coded those directly addressing the Covid‐19 pandemic (specifically, the terms corona, coronavirus, Covid‐19, or pandemic had to be mentioned in the introduction of a news item) in detail. This selection process led to an overall sample of *N* = 321 news items. Variables were based on studies by Shih et al. (2008) and Rossmann et al. (2018) and included characteristics of news coverage potentially amplifying or attenuating risk information: (1) *volume of reporting* (number of Covid‐19‐related news items per newscast), (2) *degree of debates or controversy* (none, low‐to‐medium, strong controversy), (3) *dramatization* through the use of emotional or alarming language (none, low‐to‐medium, strong), and the (4) use of potentially amplifying (*consequences* and *uncertainty*) and attenuating (*action*, *reassurance*, and *new evidence*) *media frames*. Frames were coded as either major (the frame emphasized the most throughout a news item) or minor (less prominent) frame (Rossmann et al., 2018). For each news item, one major frame had to be coded, whereas the coding of a minor frame was optional. To ensure reliable coding, each category was explained elaborately and illustrated by several examples in the codebook. Please see Table 2 for more detailed information on the different variables.

**TABLE 2:** Variables and intercoder reliabilities.

Variable	Description	Krippendorff's Alpha
Number of news items per newscast	Variable refers to the overall number of news items per newscast, regardless of the topic addressed. Based on this number, we determined the proportion of news items on Covid‐19 in relation to the overall reporting during the investigation period	0.96
Degree of debates or controversy	Describes to what extent a news item depicts different opinions or even conflicts. *No controversy* is coded when a news item presents a consensus or a single opinion. *Low‐to‐medium controversy* is coded when conflicting opinions are depicted, such as discussions among scientists or parliamentary debates. *Strong controversy* is coded when a news item depicts threats or use of violence, e.g., physical attacks at demonstrations	0.96
Dramatization	Refers to the use of alarming or emotionalizing language in a news item. *No dramatization* is coded when a news item is characterized by sober, neutral language. *Low‐to‐medium dramatization* is coded when a news item uses affective words (e.g., optimistic, forcefully, and sadly). *Strong dramatization* is coded in cases where alarming language dominates, for instance, by a pronounced use of metaphors (e.g., comparing the situation in hospitals to war zones) or superlatives	0.87
Frames	For each news item, one major frame has to be coded. The category applies if a news item is most strongly characterized by this frame. Title and introduction of a news item can provide hints. In addition to a major frame, coders may also code up to one minor frame, i.e., a frame less pronounced than the major frame but also prominent in the news item. For detailed information regarding the different frames, please see Table 1	0.81–1.0

*Note*: Variables are based on studies by Shih et al. (2008) and Rossmann et al. (2018).

With measures between 0.81 and 1.0, intercoder reliability was sufficient across all variables (Krippendorff, 2011). After pretesting and adapting the codebook, reliability measures were derived from coding *n* = 8 randomly selected newscasts (*n* = 50 news items, 15.6% of the total sample). Finally, the news items were independently coded by two trained student coders. To answer RQ1 and RQ2, we conducted several chi‐square and *t*‐tests using SPSS 29.

### 4.2 Results

With an average of 78.7% (*M* = 0.79, *SD* = 0.23) of all news items per newscast discussing the pandemic, our data show a (1) high volume of Covid‐19‐related information. Notably, in comparison to *Tagesschau*, *RTL Aktuell* addressed the topic even more often (*t*(275.45) = −12.92, *p* < 0.001). With most of the news items in the sample^2^ (67.4%) not depicting any controversy, the (2) degree of debates was low for both newscasts. This was different with regards to (3) dramatization: Only 32.2% of the news items did not contain any emotional or alarming language. However, 45.4% showed low‐to‐medium and 22.4% even strong attributes of dramatization. Importantly, the reporting of *RTL Aktuell* was more dramatizing than the reporting of *Tagesschau* (*χ* ^2^(2) = 18.449, *p* < 0.001).

**FIGURE 1:**
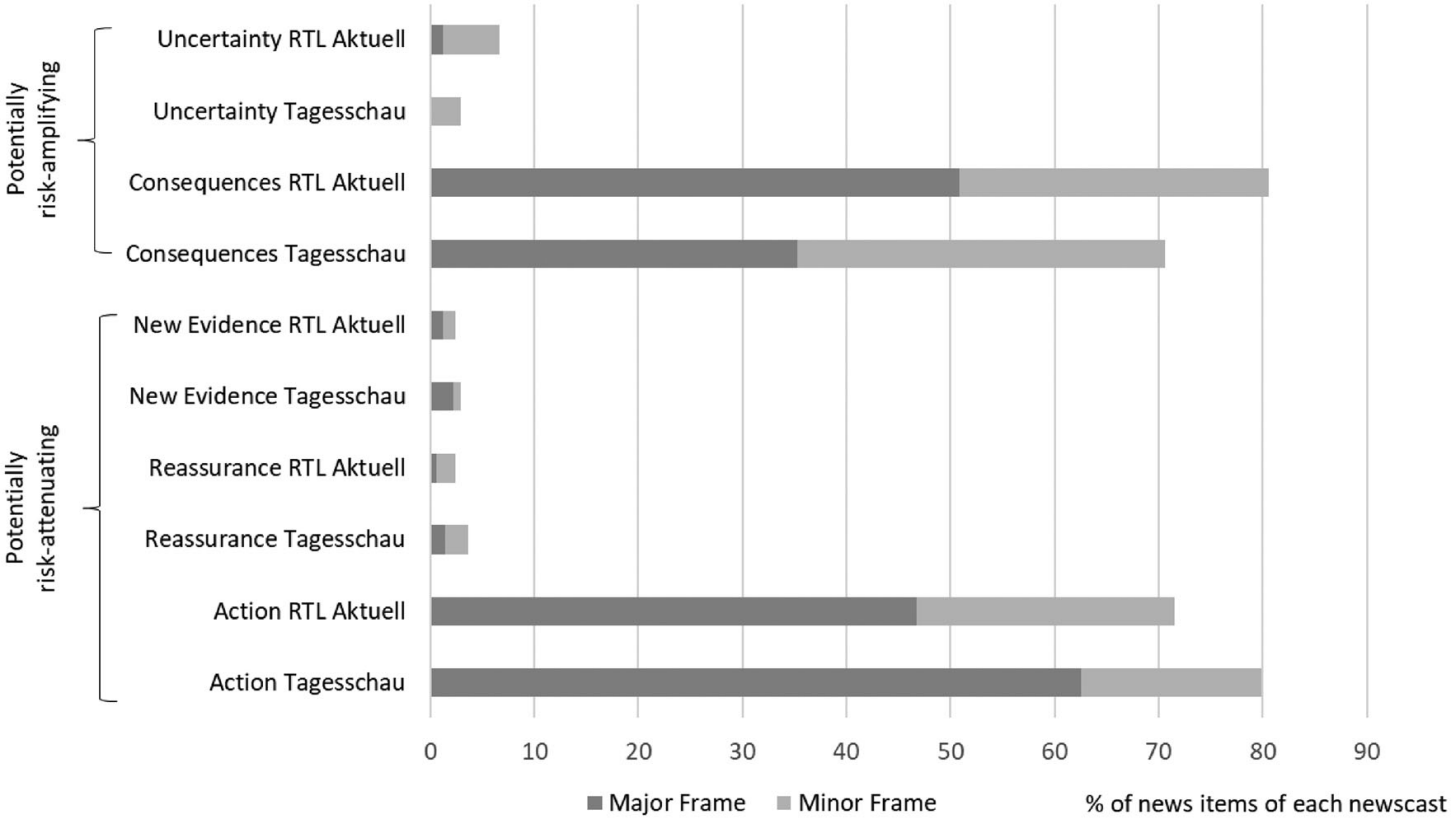
Framing of the Covid‐19 pandemic in *Tagesschau* and *RTL Aktuell*. *Note*: Percentages refer to the number of news items containing a particular risk‐attenuating, respectively, risk‐amplifying major or minor frame in relation to the number of news items of *Tagesschau* (*n* = 139), respectively, *RTL Aktuell* (*n* = 165) that were included in the frame analysis (total: *n* = 304).

As for the (4) framing of risk information (Figure 1), the (risk‐attenuating) *action* frame was the most common major frame (and second most common minor frame) throughout the sample, followed by the (risk‐amplifying) *consequences* frame, which, in turn, was the most applied minor frame. Although the *action* frame was significantly more prominent in the reporting of *Tagesschau* (*χ* ^2^(2) = 7.702, *p* = 0.021), *RTL Aktuell* laid more emphasis on the *consequences* of the pandemic (*χ* ^2^(2) = 0.156, *p* = 0.017). Notably, none of the other frames (*uncertainty*, *new evidence*, and *reassurance*) played a significant role in the Covid‐19 reporting of the two newscasts.

Overall, the TV news analyzed in our study provided both risk‐amplifying (high volume of information and dramatization, emphasis of *consequences*) and risk‐attenuating (low degree of debates, emphasis of *action*) information (RQ1). However, risk‐amplifying information were more pronounced by *RTL Aktuell* as a major private TV newscast (RQ2).

## 5 STUDY 2: PANEL SURVEY

### 5.1 Methods

We then conducted a two‐wave panel survey with a quota sample recruited by an online panel provider. Field time was between March 23 and March 31 for the first (t1; *N* = 1378) and between April 15 and April 21 for the second wave (t2; *N* = 1061), during the first Covid‐19‐related lockdown in Germany in early 2020 (Thurau & Bosen, 2021). The final sample of participants that completed both questionnaires was nearly representative for the German population regarding age (*M* = 49.0, *SD* = 15.16), gender (female: 51.2%, male: 48.8%), and education (68.0% without and 32.0% with university entrance certificate). Only a minority (8.7%) reported personal experience with the virus. All participants gave their informed consent to use and share their anonymized data for scientific purposes. In both surveys, we measured *perceived susceptibility* (“How likely do you think are you to fall ill from the coronavirus?”, 1 = not likely, 5 = very likely) and *severity* of Covid‐19 (“How serious do you think the consequences of the coronavirus would be for you personally?”, 1 = not severe at all, 5 = very severe) (Hubner & Hovick, 2020). At time 2, respondents indicated how often they had encountered Covid‐19‐related information on TV in general (1 = never, 5 = daily; information seeking *M *= 3.98, *SD* = 1.45; information scanning *M* = 4.15, *SD* = 1.32), and more specifically, how often they had received such information through public and private TV newscasts, including *Tagesschau* and *RTL Aktuell*, in between the waves. Based on these variables, we built four groups: users of both types of newscasts (*n* = 513), only public TV newscast users (*n* = 310), only private TV newscast users (*n* = 105), and non‐users (*n* = 133). We also captured further Covid‐19‐related information seeking and scanning (Lewis, 2017) between the two waves—comprising the following nine sources: medical experts, family and friends, radio, newspapers, news magazines, internet, social media, podcasts, and warning apps (1 = never, 5 = daily; information seeking *M *= 2.35, *SD* = 0.76; information scanning *M* = 2.43, *SD* = 0.77). Notably, among all the different sources, TV in general was the most used source of information, followed by family and friends (information seeking *M *= 3.43, *SD* = 1.29; information scanning *M* = 3.43, *SD* = 1.32) and internet sources (information seeking *M *= 3.22, *SD* = 1.56; information scanning *M* = 3.30, *SD* = 1.56).

To answer RQ3, we tested for significant differences in risk perceptions among the four groups at both times (ANOVAs with Duncan post hoc test). To examine if differences in risk perceptions over time can be explained by the use of TV news (RQ4), we additionally computed mixed ANOVAs for both indicators of risk perception, testing for a significant interaction of time and TV news use. In those analyses, we controlled for age and previous experience with Covid‐19 as potential determinants of risk perception (Dryhurst et al., 2020). Moreover, we controlled for Covid‐19‐related information behavior via other sources. All analyses were conducted with SPSS 29.

### 5.2 Results

In both waves, respondents in the four groups of TV news use showed significantly different Covid‐19‐related risk perceptions (RQ3; see Table 3). Respondents who did neither use public nor private TV news showed the lowest risk perception and differed significantly from all other groups.

**TABLE 3:** Mean differences in risk perceptions between groups of television (TV) news use.

	No TV news use (*n *= 133)	Only private TV news (*n *= 105)	Only public TV news (*n *= 310)	Both media (*n *= 513)	*F*‐value
Susceptibility (t1)	2.71^a^ (1.12)	3.20^b^ (1.03)	3.09^b^ (1.00)	3.04^b^ (1.00)	5.625^***^
Severity (t1)	2.66^a^ (1.19)	3.51^b^ (1.11)	3.16^c^ (1.18)	3.39^bc^ (1.18)	16.019^***^
Susceptibility (t2)	2.58^a^ (1.09)	2.84^b^ (1.04)	2.95^b^ (1.00)	2.89^b^ (1.04)	4.308^**^
Severity (t2)	2.49^a^ (1.24)	3.52^b^ (1.22)	3.04^c^ (1.20)	3.25^c^ (1.23)	17.944^***^

*Note*: Results based on ANOVAs with a Duncan post hoc test. Means are marked by different characters (a–c) and differ significantly. Perceived susceptibility (1 = not likely, 5 = very likely) and severity of Covid‐19 (1 = not severe at all, 5 = very severe).

^*^ *p* < 0.05.

^**^ *p* < 0.01.

*** *p* < 0.001.

**FIGURE 2:**
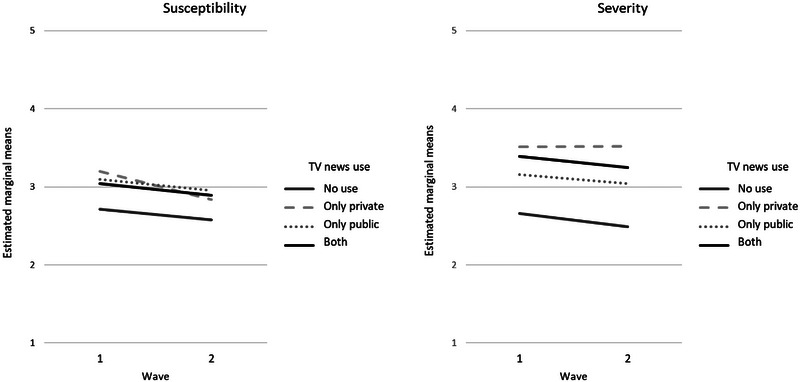
Effect of time and television (TV) news use on risk perception (age, information seeking, and information scanning as covariates).

Controlling for age, previous experience with the virus, and information behavior via other sources, there was a significant main effect of TV news use on severity (see Table 4). Moreover, there was a significant effect of time in that risk perceptions slightly decreased over time: Respondents perceived it to be less probable to get infected with Covid‐19 at wave two (susceptibility (t1) *M* = 3.03, *SD* = 1.03; susceptibility (t2) *M* = 2.87, *SD* = 1.04). Similarly, potential consequences of Covid‐19 were perceived as less severe (severity (t1) *M* = 3.25, *SD* = 1.20; severity (t2) *M* = 3.12, *SD* = 1.25). The change in perceived susceptibility can partly be attributed to TV news use because there was a significant interaction effect between time and TV news use (RQ4; see Table 4). Notably, with age and information behavior as covariates, estimated marginal means of perceived susceptibility decreased most for respondents who only used private TV news between both survey waves (see Figure 2). In contrast, there was no significant interaction of TV news use and time for severity.

**TABLE 4:** Results of the mixed ANOVA explaining risk perception at t2.

	Susceptibility				Severity			
	*F*	*df*	*p*	*η* ^2^ *p*	*F*	*df*	*p*	*η* ^2^ *p*
*Within subject*								
Time	5.953	1	0.015	0.006	8.402	1	0.004	0.008
Time × TV news use	3.359	3	0.018	0.010	0.934	3	0.423	0.003
Error(Time)		1048				1055		
*Between subject*								
Constant term	457.441	1	<0.001	0.304	54.421	1	<0.001	0.049
TV news use	1.790	3	0.147	0.005	3.698	3	0.012	0.010
Error		1048				1055		
*Control*								
Info seeking	4.837	1	0.028	0.005	11.887	1	<0.001	0.011
Info scanning	2.274	1	0.132	0.002	0.853	1	0.356	0.001
Prior experience	35.472	1	<0.001	0.033	0.049	1	0.824	0.000
Age	19.237	1	<0.001	0.018	246.099	1	<0.001	0.049

Nonetheless, the estimated marginal means point out differences among the four groups regarding the development of severity over time. Notably, although severity decreased for the other three groups, there was a slight increase in perceived severity for participants who only used private TV news between both survey waves (see Figure 2).

So, overall, our findings indicate a relation between TV news use and risk perception as the four groups of recipients differed regarding their Covid‐19‐related risk perceptions with non‐users of TV news showing the lowest risk perception, also at time 2 (RQ3). In addition, TV news (non‐)users differed in the development of risk perceptions over time, with a significant interaction effect of time and TV news use on susceptibility (RQ4).

## 6 GENERAL DISCUSSION

In our study, we addressed several gaps in literature. First, given their importance as an information source during the first stages of the Covid‐19 pandemic in Germany (Bendau et al., 2021; Dan & Brosius, 2021; Dreisiebner et al., 2022), we systematically examined the extent to which TV news exhibited characteristics that are considered to amplify, respectively, attenuate audiences’ risk perceptions. As prior studies identified media‐specific differences in the portrayal of risk information (Bhatti et al., 2022; Rossmann et al., 2018; Sandell et al., 2013)—including differences between public and private TV channels (Miconi et al., 2023)—we compared the coverage of *Tagesschau* as the most watched public TV newscast to the reporting of *RTL Aktuell* as the most watched private TV newscast (Haddad et al., 2023). Second, we were interested in the relation between TV news use and changes in audiences’ risk perception. We conducted a two‐wave panel survey among the German population in order to provide a longitudinal perspective and, in consequence, draw causal conclusions. Because news media are assumed to affect the public's risk perceptions especially in times in which people (still) lack first‐hand experience of a particular health hazard (Oh et al., 2015), our study took place during the first wave of the Covid‐19 pandemic in Germany in March and April 2020. Our data confirm low personal experience with the virus at that time.

Overall, our study shows that SARF indeed is a helpful framework for analyzing the association between the way in which the news portrays risk information and recipients’ risk perceptions. Notably, our findings offer a first integrative perspective directly linking the (1) transfer of information about the risk in TV news coverage and (2) societal response mechanisms as described by the original framework (Kasperson et al., 1988).

The results of our first study show that *Tagesschau* and *RTL Aktuell* exhibited both potentially risk‐attenuating and risk‐amplifying characteristics. The latter especially applies to the news coverage of *RTL Aktuell*, which compared to *Tagesschau* showed significantly more pronounced risk‐amplifying characteristics, including a stronger emphasis on consequences. In contrast, *Tagesschau* provided more action frames than *RTL Aktuell*, which may attenuate risk perceptions. Thus, our results confirm prior findings from the Covid‐19 pandemic in several aspects. First, they illustrate that news coverage during this major public health event was not coherent in its risk portrayal (Bhatti et al., 2022; Maurer et al., 2021) but rather characterized by both risk‐attenuating (Owusu Ansah et al., 2021) and risk‐amplifying (Ebrahim, 2022; Gering & Cohen, 2023; Hart et al., 2020) attributes. This is an important finding, because an incoherent portrayal of risk information may complicate, rather than enable, the development of adequate risk perceptions among the public during crisis events. Second, in line with prior studies (Bhatti et al., 2022; Rossmann et al., 2018; Sandell et al., 2013), we were able to identify media‐related differences in the portrayal of risk information, that is, differences between a public and a private TV channel (Miconi et al., 2023). Such channel‐related specifics should be considered when assessing the media's role as significant stations of information transfer during crisis events. More specifically, public health authorities should be aware that the way in which their communicative actions are perceived by audiences may not only depend on recipients’ personal characteristics (Zhou, 2022), but also their preferred information source.

As for our second study, our results first of all confirm the significance of TV as information source during the first wave of the Covid‐19 pandemic in Germany. Regarding audiences’ risk perceptions, we could observe a general decrease of risk perception over time and differences among the groups of TV news use. Given that Germany entered the first lockdown shortly before the start of our panel survey (Thurau & Bosen, 2021), the decrease in perceived susceptibility among all four groups (no TV news use, only private, only public, both) over time is not surprising. However, especially for non‐users, perceived susceptibility was quite low already at time 1. This may be due to comparatively low case numbers and Covid‐19‐related deaths in Germany in March 2020.^3^ Still, both had been increasing constantly and considerably since January, ultimately leading to the first lockdown (BMG, 2023).

As for the TV newscasts’ role for amplifying (or attenuating) risk perceptions, our findings indicate differences in the development of risk perceptions over time for the four groups of TV news use. Controlling for age, previous experience with the virus, and Covid‐19‐related information behavior via other sources, there was a significant interaction effect of TV news use and time on perceived susceptibility with the strongest decrease in susceptibility for those who only used private TV news—again indicating that specific characteristics of media channels should be taken into account when assessing the media's impact on risk perception during times of crisis.

In line with *RTL Aktuell*, particularly stressing consequences, of the pandemic, perceived severity was highest among those who exclusively used private TV news. However, this finding has to be interpreted with caution because there was no significant interaction effect of time and TV news use on perceived severity. Hence, it remains unclear whether private TV news served as an amplification station during the first wave of the Covid‐19 pandemic in Germany.

### 6.1 Limitations and implications for future research

Our study has several limitations. First of all, the content analysis was limited to two specific TV newscasts, namely, *Tagesschau* and *RTL Aktuell*, whereas in the survey, we assessed participants’ general use of public and/or private TV newscasts. Nevertheless, given that we selected the most successful public as well as the most successful private TV newscast, which both account for considerably high market shares (Haddad et al., 2023), the results from the content analysis and the survey can still be considered well related. However, future studies should consider a retrospective analysis of the specific news sources used by audiences to retrieve information in times of crisis. Hereby, even stronger links between survey and content‐analytic data could be provided.

Moreover, in order to capture the whole flow of risk information during a public health event such as a pandemic—from the crisis communication of authorities, to the media, and finally to audiences’ reactions—future research could additionally include, for instance, press releases of public health institutions and organizations in the analysis. In addition, experimental research is needed to determine whether the (potentially risk‐amplifying or risk‐attenuating) health crisis frames identified by Shih et al. (2008)—specifically, *action*, *reassurance*, *new evidence*, *uncertainty*, and *consequences*—do in fact influence audiences’ risk perceptions as presumed by prior research (Rossmann et al., 2018).

Regarding the absolute differences in risk perceptions among the groups of TV news use, it should be noted that we cannot draw any causal conclusions for this aspect. Possibly, people who were generally less interested in Covid‐19 and had low risk perception decided to not follow the news—rather than not using TV news, leading to low risk perceptions. However, with further analyses, we aimed at causally linking TV news use to changes in risk perception. Those analyses revealed that the interaction between time and TV news use explained 1% of the variance in participants’ perceived susceptibility and there was no significant interaction effect for severity. Given that we examined media effects assessing relationships with field survey data (and not a controlled experiment), these observed small relationships do in fact matter. Furthermore, effect sizes are often smaller in a longitudinal design as compared to cross‐sectional data, as not only the constructs in question may have changed from t1 to t2, but also many other variables playing a role. Still, this design allowed us to better mirror the assumed causal relationship between TV news use and audiences’ risk perception. Moreover, there may be other important factors influencing risk perceptions. Therefore, future research should, next to prior experience with the virus, information behavior via other sources, and sociodemographic variables, also consider additional factors, such as trust in government, science, and medical professionals (Dryhurst et al., 2020), in order to fully explain audiences’ risk perceptions. Last but not least, risk perception itself is a complex construct, and future studies may dive deeper into the dynamics between personal and general risk perceptions during a pandemic (Vieira et al., 2022).

### 6.2 Conclusions

Our study offers a first integrative perspective on risk portrayal in TV news and audiences’ responses as described by the SARF (Kasperson et al., 1988). Results indicate that through differences in the use of potentially risk‐amplifying and risk‐attenuating frames, the major public and the major private TV newscast in Germany may have influenced risk perceptions of their respective users during the first wave of the Covid‐19 pandemic. Thereby, our results also confirm media‐specific differences in the depiction of risk information during public health events such as pandemics. However, more research is needed to examine the effects of different types of media and changes in risk perceptions over time.

## CONFLICT OF INTEREST STATEMENT

The authors declare no conflicts of interest.

## FUNDING INFORMATION

There has been no financial support that could have influenced the outcome.

## ETHICS STATEMENT

This manuscript is based on two studies—a quantitative content analysis and a two‐wave panel survey. For the content analysis, no ethical approval was obtained, as we analyzed information available in the public domain and intended for a mass audience. In order to protect our two student coders from potential harm, we closely supervised them throughout the coding process. The other two studies, however, were granted ethical approval by the University of Erfurt (Application 20200312).

## Data Availability

The authors have provided the required data availability statement unless the article type is exempt, and if applicable, included functional and accurate links to said data therein.

## Footnotes

1 In their original article, Shih et al. (2008) identified one more frame, the *conflict frame*. Notably, this frame is highly similar to the *degree of debates* depicted in news coverage, as it also entails the portrayal of disputes among experts or other actors. Therefore, we chose to exclude this frame from our list.

2 In the following, percentages refer to an overall sample of *n* = 304 news items, as *n* = 17 particularly short news items by *RTL Aktuell* (“*RTL Aktuell News Kompakt*”), were excluded from further coding.

3 For instance, case numbers and Covid‐19‐related deaths were considerably higher in Italy or the United States during that time (WHO, 2020).
